# Non-linear Scaling of Passive Mechanical Properties in Fibers, Bundles, Fascicles and Whole Rabbit Muscles

**DOI:** 10.3389/fphys.2020.00211

**Published:** 2020-03-20

**Authors:** Samuel R. Ward, Taylor M. Winters, Shawn M. O’Connor, Richard L. Lieber

**Affiliations:** ^1^Department of Bioengineering, University of California, San Diego, San Diego, CA, United States; ^2^Department of Orthopaedic Surgery, University of California, San Diego, San Diego, CA, United States; ^3^Department of Radiology, University of California, San Diego, San Diego, CA, United States; ^4^School of Exercise and Nutritional Sciences, San Diego State University, San Diego, CA, United States; ^5^Veteran’s Administration San Diego Healthcare System, San Diego, CA, United States; ^6^Shirley Ryan AbilityLab, Northwestern University, Chicago, IL, United States

**Keywords:** passive tension, scaling, titin, collagen, muscle architecture

## Abstract

Defining variations in skeletal muscle passive mechanical properties at different size scales ranging from single muscle fibers to whole muscles is required in order to understand passive muscle function. It is also of interest from a muscle structural point-of-view to identify the source(s) of passive tension that function at each scale. Thus, we measured passive mechanical properties of single fibers, fiber bundles, fascicles, and whole muscles in three architecturally diverse muscles from New Zealand White rabbits (*n* = 6) subjected to linear deformation. Passive modulus was quantified at sarcomere lengths across the muscle’s anatomical range. Titin molecular mass and collagen content were also quantified at each size scale, and whole muscle architectural properties were measured. Passive modulus increased non-linearly from fiber to whole muscle for all three muscles emphasizing extracellular sources of passive tension (*p* < 0.001), and was different among muscles (*p* < 0.001), with significant muscle by size-scale interaction, indicating quantitatively different scaling for each muscle (*p* < 0.001). These findings provide insight into the structural basis of passive tension and suggest that the extracellular matrix (ECM) is the dominant contributor to whole muscle and fascicle passive tension. They also demonstrate that caution should be used when inferring whole muscle properties from reduced muscle size preparations such as muscle biopsies.

## Introduction

Passive tension is borne by a muscle when it is lengthened beyond slack length. This increased tension resists further stretch, even in the absence of muscle activation. Passive mechanical properties vary among muscles (Brown et al., 1996; Shah et al., 2004; Prado et al., 2005; Ward et al., 2009), differentiate between healthy and pathologic muscle (Abrams et al., 2000; Fridén and Lieber, 2003; Lieber et al., 2003; Meyer and Lieber, 2012), and adapt in response to altered use (Brown et al., 1996; Takahashi et al., 2007). However, understanding the underlying basis for these passive properties requires measuring passive mechanical muscle properties and identifying sources of passive tension at each size scale.

Passive tension is generally attributed to both the extracellular matrix (ECM) and intracellular titin (also known as connectin), both of which have viscoelastic properties. Muscle ECM, which is dominated by collagen in terms of mass, is organized into three interconnected levels – epimysium surrounds whole muscles, perimysium surrounds fascicles, and endomysium surrounds individual fibers (Borg and Caulfield, 1980; Trotter et al., 1995). These distinctions represent anatomical definitions with very little structural or functional basis. These various connective tissue layers provide structural support and play an important role in force transmission between fibers and tendon (Trotter and Purslow, 1992). Another passive load bearing structure is titin (Wang, 1985; Maruyama, 1986), which is a large (∼4 MDa) protein that spans from the sarcomere M-line to the Z-disk (Maruyama et al., 1985) and bears tension with increased sarcomere strain (Trombitas et al., 1995).

The relative contribution of ECM and titin may be muscle (Prado et al., 2005), size-scale (Meyer and Lieber, 2011; Smith et al., 2011), and strain dependent (Trombitas et al., 1995). Titin clearly bears passive load in myofibrils (Linke et al., 1994; Bartoo et al., 1997) and functions at low muscle strains (Wang et al., 1991; Linke et al., 1994; Trombitas et al., 1995; Brynnel et al., 2018). ECM is thought to dominate at the fiber bundle (Meyer and Lieber, 2011; Smith et al., 2011) and whole muscle (Olsson et al., 2006) level and act at high strains (Williams and Goldspink, 1984; Linke et al., 1994; Brown et al., 2011a; Meyer and Lieber, 2011). Unfortunately, most passive mechanical studies are performed at only one size scale and then extrapolated to other scales to predict structure-function relationships (Fridén and Lieber, 2003; Lieber et al., 2003; Ward et al., 2009; Brown et al., 2011a; Meyer and Lieber, 2011). With increasing size, additional layers of ECM are included in the sample, which may result in a non-linear scaling relationship in skeletal muscle. However, experimental data are not available to support or refute this concept.

In contrast to passive tension, active muscle tension clearly results from intracellular interaction among actin and myosin contractile proteins within individual sarcomeres and whole muscle active tension is accurately modeled by linearly scaling the number of sarcomeres acting in series and in parallel. At the whole muscle level, muscle architecture (i.e., the arrangement of muscle fibers within a muscle) predicts both active isometric force (Powell et al., 1984) and excursion (Winters et al., 2011) to a first approximation. Exceptions to this statement are based on higher order effects in muscles that may occur due to myofascial force transmission among muscles (Maas et al., 2001, 2003) or history-dependent changes in active force (Edman et al., 1982; Joumaa and Herzog, 2014). However, since neither myofascial force transmission nor history-dependent changes are generalizable to date, they are not typically included in biomechanical models of active muscle force. Since passive tension is mediated by both intra- and extra-cellular contributions (which are poorly understood at the various scales) scaling laws for passive muscle mechanical properties are not currently known. Indeed, our previous study of active muscle properties showed that active tension scaled linearly with sarcomere number but passive tension predictions were off by 25% to 150% depending on muscle and strain (c.f. Figure 3 of reference Winters et al., 2011).

Thus, the purpose of this study was to measure the passive mechanical properties of single muscle fibers, fiber bundles, fascicles, and whole muscles from the same samples of three architecturally diverse muscles from New Zealand White rabbits. Fiber number, titin isoform, and collagen content were also measured to provide insights into, which, if any of these parameters affect passive modulus at each scale.

## Materials and Methods

### Whole Muscle Mechanical Testing

All experimental procedures were approved by the Institutional Animal Care and Use Committee (IACUC) of the University of California, San Diego. The tibialis anterior (TA; *n* = 6), extensor digitorum longus (EDL; *n* = 6), and extensor digitorum of the second toe (ED2; *n* = 6) muscles of New Zealand White rabbits (mass = 2.68 ± 0.04 kg; mean ± standard error of the mean) were chosen due to their highly varied architecture and accessibility (Lieber and Blevins, 1989; Takahashi et al., 2007). Animal preparation and measurement of whole muscle isometric contractile properties were performed as previously described (Lieber et al., 1991; Lieber and Friden, 1993). Briefly, rabbits were anesthetized using a subcutaneous injection of a ketamine-xylazine cocktail (35 and 5 mg/kg, respectively) and maintained on 2% isoflurane (IsoFlo; Abbott Laboratories, North Chicago, IL, United States) at 2 L/min through a face-mask. Heart rate and oxygen saturation were monitored (VetOx, Heska Co., Fort Collins, CO, United States) throughout the test. A mid-line incision was made from the mid-thigh to the ankle to expose the muscle and tendon. The hindlimb was immobilized in a custom jig via tightened screws at the femoral condyles and malleoli. Suture markers were placed at the distal and proximal muscle-tendon junctions to define muscle length (L_m_). The distal tendon was transected, released from the extensor retinaculum (for TA and EDL), and clamped to a servomotor (Cambridge Model 310B; Aurora Scientific, Aurora, ON, Canada) at the muscle-tendon junction and aligned with the force-generating axis of the motor (Lieber et al., 1991). For TA and EDL testing, both tendons were cut to avoid the confounding effect of the other muscle.

The mechanical testing protocol consisted of a series of passive stretches over a range of muscle lengths. Neutral muscle length (L_m__0_) was defined as the muscle length at which the hip and knee were at 90° and the ankle was at 0° dorsiflexion and neutral, muscle fiber length (L_f__0_) was calculated using previously determined fiber length-to-muscle length ratios (Lieber and Blevins, 1989). Measurements were obtained at discrete lengths ranging from L_m__0_−5% L_f__0_ to L_m__0_+40% L_f__0_ in increments of 5% L_f__0_ with surrounding muscles maintained at their *in situ* length. Passive tension at each length was measured 3-min after each stretch to permit stress-relaxation. Force and length were acquired at 4 kHz (610E series; National Instruments, Austin, TX, United States) and a custom-written LabView program (National Instruments). This sequence was repeated for each of the three muscles in each animal.

Upon completion of testing, animals were euthanized with pentobarbital (Euthasol; Virbac AH, Fort Worth, TX, United States) and muscles were removed, blotted dry and weighed. Muscles were then divided longitudinally with one half (randomized) fixed at neutral length for 3–5 days in 10% buffered formalin for architectural determination. The distal one-third of the other section was removed and placed into a glycerinated storage solution [composed of 170.0-mM KPropionate, 5.0-mM K_3_EGTA, 5.3-mM MgCl_2_, 10.0-mM imidazole, 21.2-mM Na_2_ATP, 1.0-mM NaN_3_, 2.5-mM glutathione, 50-μM leupeptin, and 50% (v/v) glycerol] at −20°C for up to 4 weeks (Fridén and Lieber, 2003; Lieber et al., 2003; Einarsson et al., 2008) for further *in vitro* passive mechanical testing.

### Fiber, Fiber Bundle, and Fascicle Mechanical Testing

**FIGURE 1:**
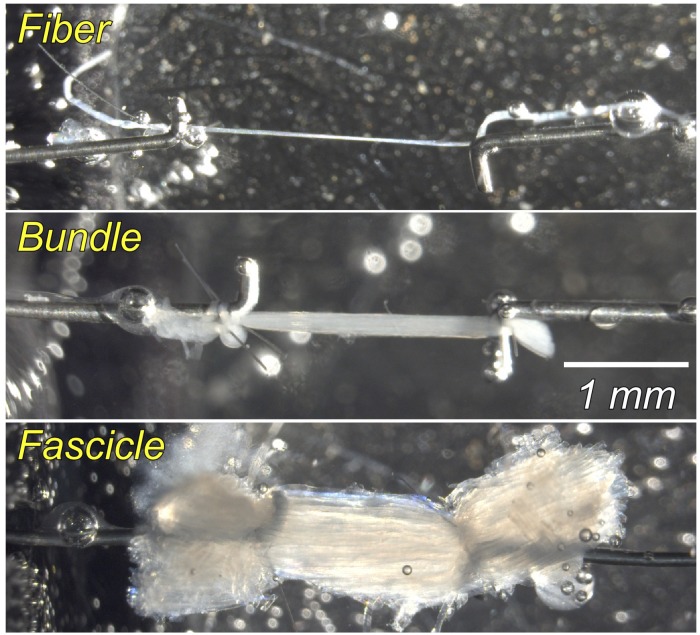
Photomicrograph of samples at the single fiber, fiber bundle (approximately 20 fibers), and fascicle (∼300 fibers, defined by natural ECM divisions) scale. Each sample (1.5–3.0 mm in length) was secured using suture to a force transducer on one end and a titanium wire rigidly attached to a rotational bearing on the other end. Each stretch was held for 3 min to permit stress-relaxation before measuring passive tension. See text for details.

Muscles were removed from storage solution and transferred into chilled relaxing solution maintained at pCa 8.0 and pH 7.1, consisting of the following: 59.4-mM imidazole, 86.0-mM KCH_4_O_3_S, 0.13-mM Ca(KCH_4_O_3_S)_2_, 10.8-mM Mg(KCH_4_O_3_S)_2_, 5.5-mM K_3_EGTA, 1.0-mM KH_2_PO_4_, 5.1-mM Na_2_ATP, and 50.0-μM leupeptin. Single fibers, fiber bundles (approximately 20 fibers), and fascicles (approximately 300 fibers defined by natural ECM divisions) were carefully dissected from the same muscle. Each sample (1.5–3.0 mm in length) was mounted in a chamber in a custom apparatus at room temperature (20°C). Using 10-0 monofilament nylon suture, samples were secured to a force transducer (Model 405A for fibers and bundles and 404A for fascicles, Aurora Scientific, Ontario, Canada) on one end and to a titanium wire rigidly attached to a rotational bearing (Newport MT-RS; Irvine, CA, United States) on the other end (see Figure 1 for sample illustrations of each size scale). Segments displaying obvious abnormalities or discolorations were not used. Samples were transilluminated by a low-powered laser diode to permit sarcomere length measurement by laser diffraction (Lieber et al., 1984).

Each segment was brought to slack length (length at which tension was ∼2 μN), and segment diameter and length measured optically with a cross-hair reticule mounted on a dissecting microscope. Samples were deformed in incremental strains of ∼0.25 μm/sarcomere and each stretch was held for 3 min to permit stress relaxation before measuring passive tension (Fridén and Lieber, 2003). Segments were stretched in total to ∼100% strain. Samples were discarded if they did not produce a clear diffraction pattern, if irregularities appeared along their length during testing, or if they were disrupted at either suture attachment site. Two fibers, bundles, and fascicles from different regions of each muscle were tested to avoid regional bias. After testing, samples were transferred to microcentrifuge tubes and half was suspended in sodium dodecyl sulfate-vertical agarose gel electrophoresis (SDS-VAGE) sample buffer (Warren et al., 2003) for titin analysis and the other half placed in an empty tube for collagen content analysis. All samples were stored at −80°C until biochemical analysis.

### Titin Gels

Titin molecular mass was quantified in each specimen using sodium dodecyl sulfate-vertical agarose gel electrophoresis (SDS-VAGE), as described previously (Warren et al., 2003). Briefly, samples (∼5-mg wet weight) were homogenized, then boiled for 2 min in 5:1 SDS-VAGE sample buffer. Titin standards from human soleus (3,716 kDa) and rat cardiac muscle (2,992 kDa) (Labeit and Kolmerer, 1995; Freiburg et al., 2000) were also homogenized and mixed to create a titin standards “cocktail” with the following ratio: 1-unit human soleus: 3-units rat cardiac: 6-units SDS-VAGE sample buffer. Four standard lanes, containing the human soleus and rat cardiac homogenates, were evenly distributed across the gel. Sample wells were then loaded with both experimental and rat cardiac homogenate. Gels were electrophoresed on a dual slab gel chamber (C.B.S. Scientific, Del Mar, CA, United States) at 4°C for 5 h at 25-mA constant current.

Gels were fixed, allowed to dry for ∼20 h at 40°C, and silver stained according to the Silver Stain Plus procedure (Bio-Rad, Hercules, CA, United States). Relative mobility and intensity of each band were quantified with a densitometer (GS-800, Bio-Rad) and Quantity One 1-D Analysis software (Bio-Rad). Molecular mass of each experimental band was calculated based on relative mobility compared to the human soleus and rat cardiac titin standards. As TA contained two bands, mass was calculated by averaging the two bands weighted by their relative density (Thacker et al., 2011).

### Collagen Content

Collagen percentage was determined by colorimetric analysis of hydroxyproline content (10). Briefly, samples were hydrolyzed in 6-N HCl for 24 h at 110°C, dried, and treated with chloramine T solution for 20 min at room temperature followed by a solution of *p*-diaminobenzaldehyde for 30 min at 60°C. Sample absorbance was read at 550 nm in triplicate and compared to a standard curve to quantify hydroxyproline content. Hydroxyproline content was converted to collagen percentage using the constant (7.14; Etherington and Sims, 1981) that defines the frequency of hydroxyproline residues per mole of collagen and then normalized to specimen wet weight to obtain collagen percentage in units of μg collagen/100 μg of wet weight (Edwards and O’brien, 1980).

### Muscle Architecture

Muscle architecture was quantified according to the methods of Sacks and Roy (1982) as modified in by Lieber and Blevins (1989) to measure muscle and fiber length, pennation angle, and sarcomere length. Muscle halves were removed from 10% formalin, rinsed in isotonic phosphate-buffered saline, and digested in 15% H_2_SO_4_ for 20 min to facilitate muscle fiber bundle microdissection. Two fiber bundles from three regions (six total) of each muscle were microdissected to measure fiber length and then sarcomere length (L_s_) was measured by laser diffraction (Lieber et al., 1984). Muscle length and fiber length were normalized to a sarcomere length of 2.5 μm to facilitate comparison among muscles.

To define muscle anatomical range, three separate New Zealand White rabbits were anesthetized with isoflurane and sacrificed via pentobarbital injection. Their hindlimbs were skinned and amputated mid-thigh. Three hindlimbs were pinned onto a corkboard at their maximal anatomic dorsiflexion with toes fully extended (i.e., the longest anatomical length) and three hindlimbs pinned at their maximum anatomic plantarflexion with toes fully flexed (i.e., the shortest anatomical length). Pinned hindlimbs were fixed in 10% buffered formalin for 48 hours and muscle architecture determined. Sarcomere length operating range of each muscle was determined by subtracting average L_s_ of each muscle in full dorsiflexion from average L_s_ in full plantarflexion.

### Data Analysis

From whole muscle experiments, force was converted to stress by normalizing to the muscle’s physiological cross-sectional area (PCSA). PCSA (cm^2^) was calculated according to the equation [M∙cos(θ)]/(L_fn_∙ρ) where M is measured muscle mass, θ is pennation angle, L_fn_ is fiber length normalized to sarcomere length from muscle architecture, and ρ is muscle density of 1.0597 g/cm^3^ (Mendez and Keys, 1960). For fibers, bundles, and fascicles, force data were converted to stress by dividing force by resting geometrical cross-sectional area determined assuming a cylindrical sample with average diameter determined from 3 separate points along the specimen. In all cases, length was converted to strain by normalizing to specimen slack length.

Passive stress-strain curves of muscle fibers were highly linear and fit using linear regression (*r*^2^: = 0.93 ± 0.03 across muscles). For fibers, we used linear fits, because even slight deviations from linearity using polynomial fits resulted in negative moduli when tangent modulus was calculated as the derivative of the stress-strain curve. For fibers, linear fits were equally as accurate as polynomial fits (polynomial fits yielded *r*^2^: = 0.95 ± 0.02 across muscles) so linear fits were selected. Passive stress-strain curves of larger specimens were highly non-linear and thus fit using a second-order polynomial (*r*^2^: = 0.98 ± 0.01 across scales and muscles). For these bundles and muscles, linear fits were highly inaccurate (linear fits yielded *r*^2^ values ranging from 0.3 to 0.6 across muscles) so second order polynomial fits were selected for bundles and muscles. To test this overall analytical approach, modulus data were compared to modulus calculated by direct differentiation of stress-strain data and they agreed well demonstrating that the results were not influenced by the curve-fitting approach.

Muscle and size scale were compared by two-way ANOVA with repeated measures (using grouping factors of muscle and scale), and pair-wise comparisons with LSD corrections used to compare all levels (SPSS, Chicago, IL, United States). Significance level was set to *p* < 0.05, and data are presented in text and figures as mean ± SEM (standard error of the mean).

## Results

Whole muscle architectural parameters and single fiber, fiber bundle, and fascicle dimensions were measured at each scale for the TA, EDL, and ED2 (Table 1). Specimen diameter and slack sarcomere length were not significantly different among TA, EDL, and ED2 for any size scale (Table 1). However, *in vivo* sarcomere length operating range was significantly different among muscles (Table 1; *p* < 0.05).

**TABLE 1:** Sample dimensions.

		TA	EDL	ED2
Single fiber	Diameter [mm]	0.065 ± 0.005	0.072 ± 0.005	0.064 ± 0.004
	Slack sarcomere length [μm]	2.25 ± 0.09	2.33 ± 0.10	2.36 ± 0.03
Fiber bundle	Diameter [mm]	0.267 ± 0.018	0.239 ± 0.026	0.248 ± 0.017
	Slack sarcomere length [μm]	2.32 ± 0.04	2.34 ± 0.05	2.26 ± 0.08
Fascicle	Diameter [mm]	1.198 ± 0.126	1.308 ± 0.213	0.974 ± 0.176
	Slack sarcomere length [μm]	2.22 ± 0.08	2.37 ± 0.04	2.38 ± 0.07
Whole muscle	Dorsiflexion sarcomere length [μm]	2.37 ± 0.18	2.43 ± 0.21	2.77 ± 0.03^*,#^
	Plantarflexion sarcomere Length [μm]	2.95 ± 0.07^#^	3.61 ± 0.04*	2.94 ± 0.10^#^
	Normalized fiber length [mm]	38.41 ± 1.60^#^	15.43 ± 0.97*	10.64 ± 0.82^*,#^
	Pennation angle [°]	0.6 ± 0.2^#^	8.7 ± 0.8*	18.1 ± 2.9^*,#^
	PCSA [cm^2^]	0.781 ± 0.029^#^	1.927 ± 0.117*	0.620 ± 0.064^*,#^

*Statistically significant different from TA; ^#^Statistically significant different from EDL.

**FIGURE 2:**
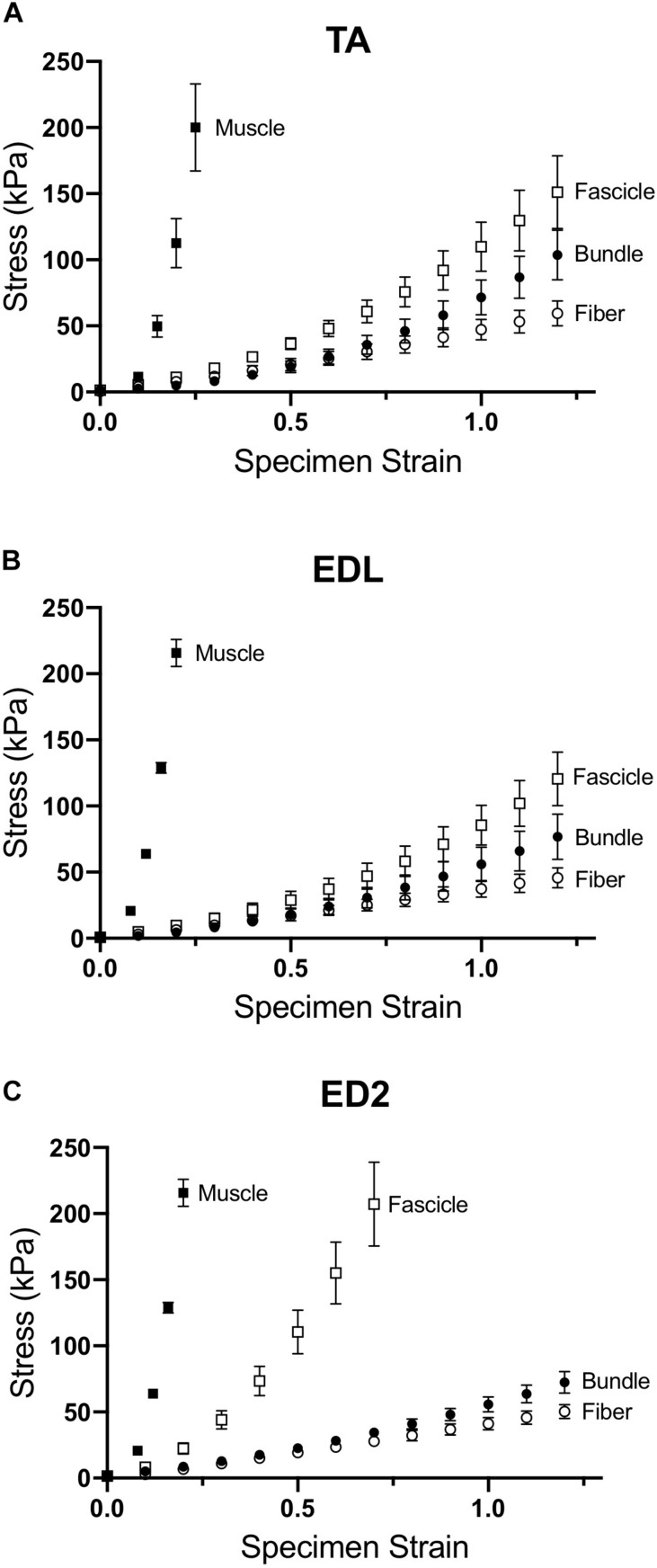
Passive stress-strain curves for **(A)** TA, **(B)** EDL, and **(C)** ED2 with each curve representing a different size scale: fibers (open circles), bundles (filled circles), fascicles (open squares), and whole muscle (filled squares). As size scale increased, samples increased in stiffness and became highly non-linear, presumably due to changes in ECM. Data are presented as mean ± SEM.

**FIGURE 3:**
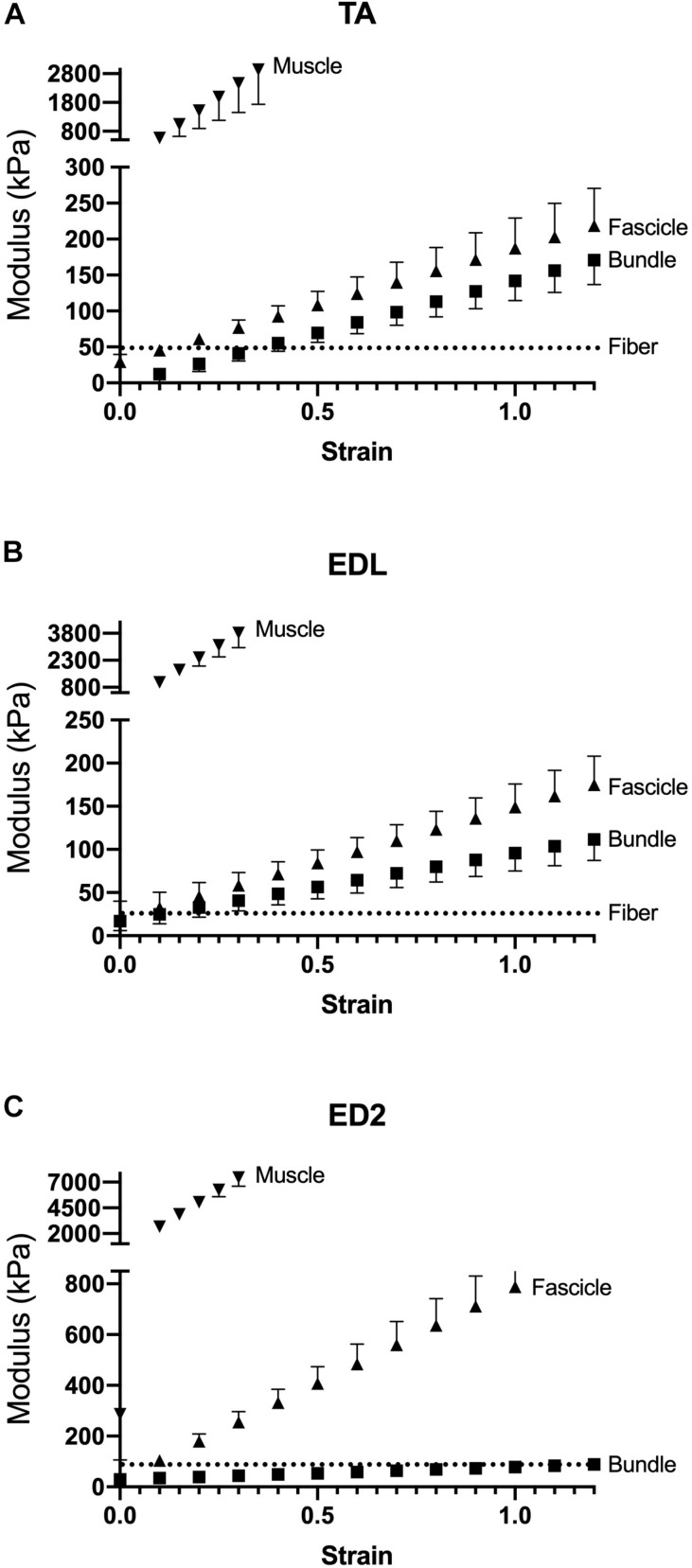
Passive modulus-strain curves for **(A)** TA, **(B)** EDL, and **(C)** ED2 with each curve representing a different size scale: fibers (dotted lines), bundles (squares), fascicles (triangles), and whole muscle (inverted triangles). As size scale increased, modulus increases. Data are presented as mean ± SEM.

Measured stress increased for a given strain for all three muscles as scale increased (Figure 2). For the TA and EDL, fascicles, bundles and fibers clustered together compared to the very high stress observed for the whole muscles (Figures 2A,B). Interestingly, the ED2 fascicles were intermediate in their stress-strain relationship between the fiber, bundle and whole muscle scales (Figure 2C). These relationships became more obvious upon examining the passive modulus, which increased with increasing size scale (*p* < 0.0001; Figure 3) and was significantly different among muscles (*p* < 0.0001). Importantly, modulus at each scale was muscle dependent as evidenced by the highly significant muscle × scale interaction term (*p* < 0.0001). There were relatively small differences in modulus between fibers and bundles. The most dramatic modulus differences occurred between the fascicle and whole muscle levels where muscles were from 5- to 10-fold higher in modulus compared to fascicles and from 10- to 100-fold higher in modulus compared to fibers. These results explicitly demonstrate that passive modulus varies across the three muscles and size scale.

**FIGURE 4:**
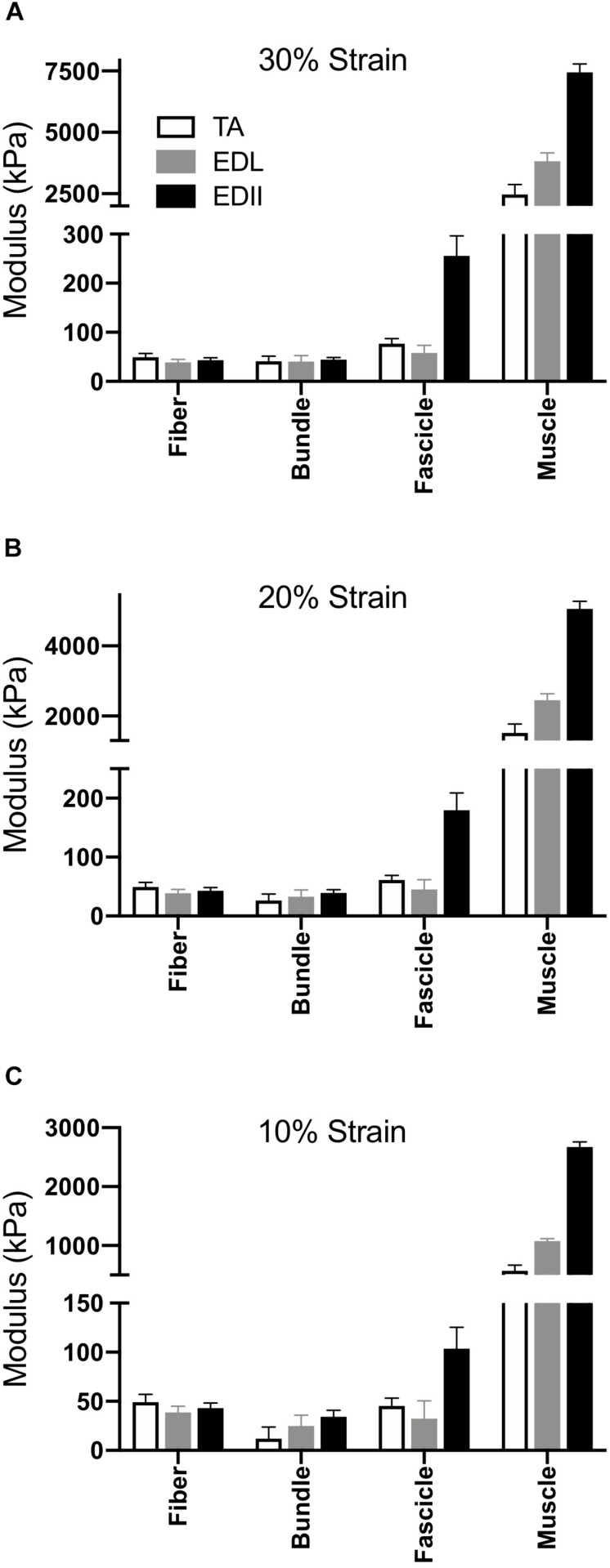
Scaling of passive tension modulus across four size scales and three muscles. **(A)** Modulus calculated at 30% strain, **(B)** Modulus calculated at 20% strain, **(C)** Modulus calculated at 10% strain. In all cases, two-way ANOVA revealed a significant effect of scale and muscle with significant scale × muscle interaction (*p* < 0.0001).

**FIGURE 5:**
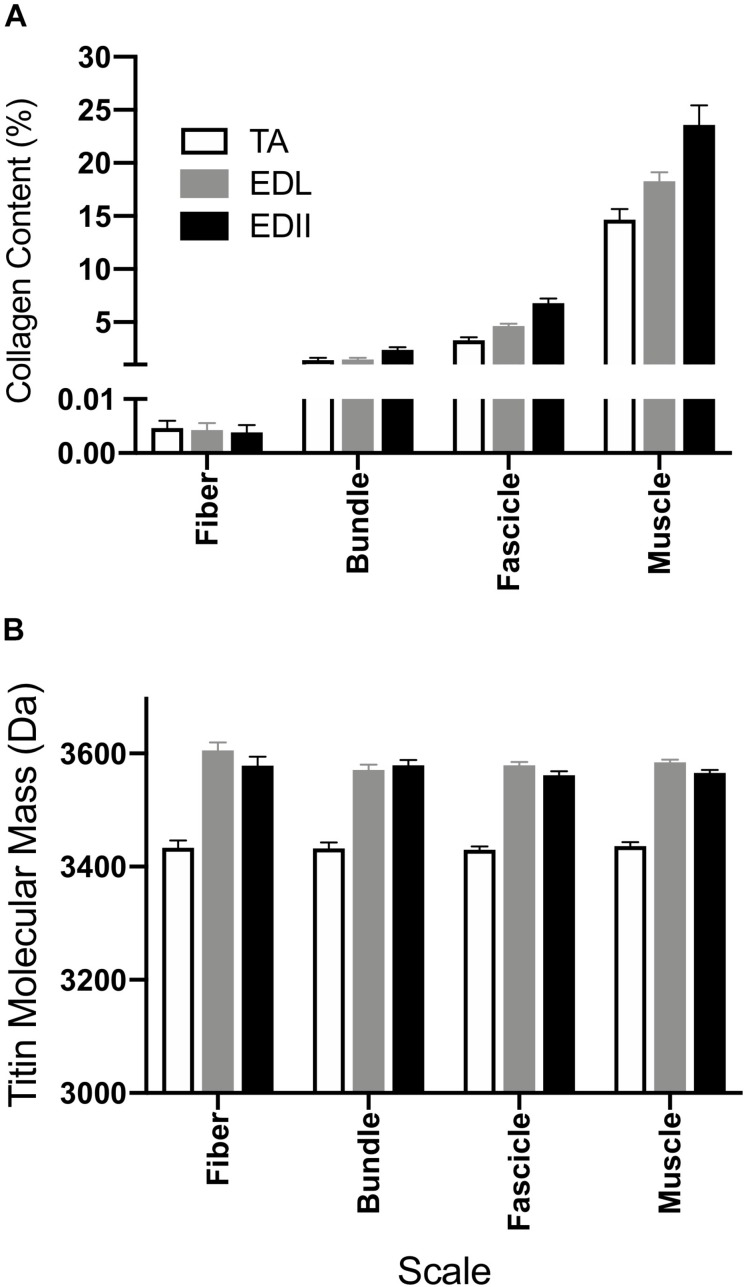
Measurement of muscle biochemical content to provide insights into the sources of passive tension. **(A)** Average collagen content was determined for fibers, bundles, fascicles, and whole muscle homogenate using a hydroxyproline assay. Collagen content increased with size scale (*p* < 0.0001) and was significantly different across muscles (*p* < 0.0001) with significant scale × muscle interaction (*p* < 0.0001). Collagen content is expressed as a percentage of wet weight. **(B)** Average titin molecular weight is shown for fibers, bundles, and fascicles and a whole muscle homogenate from TA, EDL, and ED2. Titin molecular mass was not significantly different across size scales for any of the muscles tested (*p* = 0.24). However, titin isoform size for TA was significantly smaller compared to EDL and ED2 (*p* < 0.0001). Data are presented as mean ± SEM.

Given that typical muscle sarcomere strains average about 20% (Burkholder and Lieber, 2001), we quantified modulus at strains of 10%, 20%, and 30% (Figure 4). The main result was the same for any strain – modulus was significantly different among scales (*p* < 0.0001) and muscles (*p* < 0.0001) with a highly significant muscle × scale interaction term (*p* < 0.0001). This result explicitly demonstrates the dependence of muscle passive mechanical properties on both the scale of the specimen and the specific muscle studied. Importantly, none of the conclusions of this report depend on a single strain measurement either during a functional movement or anatomical limitation. The data presented across very large strain ranges (Figure 3) and several select strain ranges (Figure 5) provide ample quantitative values to be used in future modeling studies.

To further quantify scaling rules, modulus was compared against the estimated number of fibers in each specimen on a log-log plot. Data were accurately fit using a simple power law equation (*y* = ax^b^; TA: *r*^2^ = 0.84; EDL: *r*^2^ = 0.93; ED2: *r*^2^ = 0.92), which further illustrates the non-linear scaling relationship that occurs across size scales. Furthermore, the TA exhibited a different scaling pattern than EDL or ED2 based on the different exponent value extracted from the power law (b in *y* = ax^b^; TA = 0.23 ± 0.02; EDL = 0.37 ± 0.02; ED2 = 0.41 ± 0.02; *p* < 0.001).

To provide structural insight into the differential passive tension results described above, titin molecular mass and collagen content of each specimen were measured (Figure 5). Collagen content increased significantly with scale (*p* < 0.0001), was significantly different across muscles (*p* < 0.0001) and showed a significant muscle × scale interaction (*p* < 0.0001; Figure 5A), suggesting differences in collagen organization among these three muscles. Titin molecular mass did not differ significantly across scale for any muscle tested (*p* = 0.24; Figure 5B), but did vary by muscle (*p* < 0.0001) with TA titin size significantly smaller than either EDL or ED2 at all scales (*p* < 0.001). No significant scale x muscle interaction was observed (*p* = 0.49).

## Discussion

The results of these experiments demonstrate two important concepts. First, passive modulus is not a constant for muscle that can be easily applied across scales. Second, modulus increases across scales in a non-linear, muscle-specific manner, emphasizing that extracellular sources of passive tension dominate at the whole muscle level.

Magid and Law (1985) first demonstrated that frog muscle fibers and whole muscles have the same passive modulus, demonstrating that passive tension was mediated intracellularly across scale in frog muscle. This finding led to spectacular experiments that have elucidated the nature of the intracellular titin protein. However, more current findings suggest that their finding may be species specific (Meyer and Lieber, 2018). Human muscle fibers demonstrate different passive mechanical properties compared to bundles, which contain a connective tissue matrix layer (i.e., perimysium) that individual fibers do not (Fridén and Lieber, 2003; Lieber et al., 2003; Ward et al., 2009). Differences between fibers and bundles manifest themselves functionally as well in mouse (Meyer and Lieber, 2011, 2018), rat (Brown et al., 2011a), and rabbit (Brown et al., 2011b) skeletal muscle: bundles exhibit a larger stress at a given strain that is non-linear, whereas individual fibers often demonstrate a highly linear response (Figure 2). In our sample, linear fits to fiber stress-strain data explained over 90% of the experimental variability. By comparing a group of dissected individual fibers to a fiber bundle, the non-linearity and modulus increase was shown to be due to the properties of the ECM (Purslow and Trotter, 1994; Meyer and Lieber, 2011). Here, we found that the non-linearity and magnitude of stress-strain curves increase considerably across the fiber, bundle, fascicle, and whole muscle size scales (Figure 2) and that passive modulus correspondingly increases with size scale. Fiber passive modulus measured only 3.2%, 1.6% or 0.85% of the whole muscle values for TA, EDL, and ED2, respectively, at 20% strain (Figure 4B) demonstrating that whole muscle passive tension is dominated by extracellular structures.

It was also interesting to note that the ED2, with the very high fascicle and muscle stiffness (Figure 4), had an extremely small anatomical sarcomere length range (6.1% strain, 0.17 μm; Table 1) suggesting that it probably functions over a very narrow sarcomere length range. This may suggest that it acts as a passive strut (stabilizer) rather than a muscle that moves limbs over a wide range of motion. The TA, with the lowest stiffness is monoarticular and likely functions over the largest range during normal ankle rotation (24% strain, 0.58 μm; Table 1). The EDL with intermediate stiffness may function over a relatively small range since ankle and digital rotation during normal movement are often complementary, even though it’s potential anatomical range is quite large (49% strain, 1.18 μm; Table 1). None of these interpretations are confounded by sarcomere length differences across size scales which were all very close to 2.5 μm. Future studies measuring fiber and sarcomere length during movement are necessary to explicitly test these hypotheses. Muscle fiber activation would certainly shorten sarcomeres and the anatomical studies performed herein do not necessarily emulate sarcomere length changes during movement.

It is interesting that the passive moduli of whole muscles were an order of magnitude larger than fascicles, suggesting either the fascicles tested contained an incomplete perimysium or that the epimysium is dramatically stiffer than the other ECM layers. Thus, there may be a level of connective tissue organization above fascicles that is not appreciated based on current concepts of muscle structure. Unfortunately, this presents a challenge to further clarification as there are currently not tools available that can measure muscle structure with micron resolution across distances of many millimeters. We recently reported the existence of perimysial cables of this dimension (Gillies et al., 2017) but these data are currently only obtainable on fixed/embedded specimens through the very tedious serial blockface electron microscopic method (Gillies et al., 2014).

Titin is thought to be the primary determinant of passive stiffness (Prado et al., 2005) and slack sarcomere length (Wang et al., 1991) at small size scales such as myofibrils and fibers. Titin isoforms in TA were smaller than in EDL or ED2 (Figure 5B), and, while not significant, TA did have a shorter slack sarcomere length compared to EDL and ED2 (Table 1) which may be related. Our results in rabbit muscle are in contrast to the recent report by Brynnel et al. (2018) who showed that whole mouse diaphragm, soleus and EDL muscle stiffness were all increased by the same amount when the PEVK region of titin was reduced in size by homologous recombination. Deletion of 47 exons in this region was predicted to shorten the titin filament by 63 nm which agreed well with the measured shift in the length-tension curve and increase in passive stiffness. Currently, it is not clear whether differences between these studies are species-specific or another as yet unidentified structural property of skeletal muscle.

Inspection of the biomechanical modeling literature reveals that passive mechanical properties of muscle are nearly universally considered to scale to muscle architecture properties. The definitions used for passive mechanical properties vary markedly across the numerous studies (see Table 1 of reference Meyer and Lieber, 2018). However, the data reported here clearly demonstrate that passive modulus scales in a highly non-linear fashion from fiber to whole muscle, and that the passive scaling is muscle-specific so that generic generalization across muscles is not justified. This also suggests that extrapolating whole muscle function from reduced specimen preparations may not be justified. We previously pointed this out based on the finding that active muscle force was easily predicted based on known sarcomere and architectural properties whereas passive force was not (44). In addition, the structures responsible for mediating passive tension may be fundamentally different across size scales and strains. While titin is the most significant predictor of passive tension at small size scales (e.g., myofibrils and fibers), collagen content is the strongest predictor of whole muscle passive function at any strain. These findings suggest that a fundamental difference exists among fibers, bundles, fascicles, and whole muscles, and investigators should not simply rely on reduced preparations such as muscle biopsies to infer whole muscle passive mechanical properties.

## Disclosure

The authors confirm that the publication of this paper involves no conflicts of interest of any kind.

## Data Availability Statement

The datasets generated for this study are available on request to the corresponding author.

## Ethics Statement

The animal study was reviewed and approved by the University of California, Institutional Animal Care and Use Committee (IACUC).

## Author Contributions

All authors listed have made a substantial, direct and intellectual contribution to the work, and approved it for publication.

## Conflict of Interest

The authors declare that the research was conducted in the absence of any commercial or financial relationships that could be construed as a potential conflict of interest.

## References

[B1] Abrams R. A. Tsai A. M. Watson B. Jamali A. Lieber R. L. (2000). Skeletal muscle recovery after tenotomy and 7-day delayed muscle length restoration. *Muscle Nerve* 23 707–714. 10.1002/(sici)1097-4598(200005)23:5<707::aid-mus7>3.0.co;2-t 10797393

[B2] Bartoo M. L. Linke W. A. Pollack G. H. (1997). Basis of passive tension and stiffness in isolated rabbit myofibrils. *Am. J. Physiol.* 273 C266–C276. 9252465 10.1152/ajpcell.1997.273.1.C266

[B3] Borg T. K. Caulfield J. B. (1980). Morphology of connective tissue in skeletal muscle. *Tissue Cell* 12 197–207. 10.1016/0040-8166(80)90061-0 7361300

[B4] Brown I. E. Liinamaa T. L. Loeb G. E. (1996). Relationships between range of motion, lo, and passive force in five strap-like muscles of the feline hind limb. *J. Morphol.* 230 69–77. 10.1002/(sici)1097-4687(199610)230:1<69::aid-jmor6>3.0.co;2-i 8843689

[B5] Brown S. H. Carr J. A. Ward S. R. Lieber R. L. (2011a). Passive mechanical properties of rat abdominal wall muscles suggest an important role of the extracellular connective tissue matrix. *J. Orthop. Res.* 30 1321–1326. 10.1002/jor.22068 22267257 PMC3337947

[B6] Brown S. H. Gregory D. E. Carr J. A. Ward S. R. Masuda K. Lieber R. L. (2011b). ISSLS prize winner: adaptations to the multifidus muscle in response to experimentally induced intervertebral disc degeneration. *Spine* 36 1728–1736. 10.1097/BRS.0b013e318212b44b 21301396

[B7] Brynnel A. Hernandez Y. Kiss B. Lindqvist J. Adler M. Kolb J. (2018). Downsizing the molecular spring of the giant protein titin reveals that skeletal muscle titin determines passive stiffness and drives longitudinal hypertrophy. *Elife* 7:e40532. 10.7554/eLife.40532 30565562 PMC6300359

[B8] Burkholder T. J. Lieber R. L. (2001). Sarcomere length operating range of muscles during movement. *J. Exp. Biol.* 204 1529–1536. 11296141 10.1242/jeb.204.9.1529

[B9] Edman K. A. P. Elzinga G. Noble M. I. M. (1982). Residual force enhancement after stretch of contracting frog single muscle fibers. *J. Gen. Physio.* 80 769–784. 10.1085/jgp.80.5.769 6983564 PMC2228643

[B10] Edwards C. A. O’brien W. D. Jr. (1980). Modified assay for determination of hydroxyproline in a tissue hydrolyzate. *Clin. Chim. Acta* 104 161–167. 10.1016/0009-8981(80)90192-8 7389130

[B11] Einarsson F. Runesson E. Friden J. (2008). Passive mechanical features of single fibers from human muscle biopsies–effects of storage. *J. Orthop. Surg. Res.* 3:22. 10.1186/1749-799X-3-22 18538032 PMC2432050

[B12] Etherington D. J. Sims T. J. (1981). Detection and estimation of collagen. *J. Sci. Food Agric.* 32 539–546. 10.1002/jsfa.2740320603

[B13] Freiburg A. Trombitas K. Hell W. Cazorla O. Fougerousse F. Centner T. (2000). Series of exon-skipping events in the elastic spring region of titin as the structural basis for myofibrillar elastic diversity. *Circ. Res.* 86 1114–1121. 10.1161/01.res.86.11.1114 10850961

[B14] Fridén J. Lieber R. L. (2003). Spastic muscle cells are shorter and stiffer than normal cells. *Muscle Nerve* 27 157–164. 10.1002/mus.10247 12548522

[B15] Gillies A. R. Bushong E. A. Deerinck T. J. Ellisman M. H. Lieber R. L. (2014). Three-dimensional reconstruction of skeletal muscle extracellular matrix ultrastructure. *Microsc. Microanal.* 20 1835–1840. 10.1017/S1431927614013300 25275291 PMC4267978

[B16] Gillies A. R. Chapman M. A. Bushong E. A. Deerinck T. J. Ellisman M. H. Lieber R. L. (2017). High resolution three-dimensional reconstruction of fibrotic skeletal muscle extracellular matrix. *J. Physiol.* 595 1159–1171. 10.1113/JP273376 27859324 PMC5309386

[B17] Joumaa V. Herzog W. (2014). Calcium sensitivity of residual force enhancement in rabbit skinned fibers. *Am. J. Physiol. Cell Physiol.* 307 C395–C401. 10.1152/ajpcell.00052.2014 24965591 PMC4137138

[B18] Labeit S. Kolmerer B. (1995). Titins: giant proteins in charge of muscle ultrastructure and elasticity. *Science* 270 293–296. 10.1126/science.270.5234.293 7569978

[B19] Lieber R. L. Blevins F. T. (1989). Skeletal muscle architecture of the rabbit hindlimb: functional implications of muscle design. *J. Morphol.* 199 93–101. 10.1002/jmor.1051990108 2921772

[B20] Lieber R. L. Friden J. (1993). Muscle damage is not a function of muscle force but active muscle strain. *J. Appl. Physiol.* 74 520–526. 10.1152/jappl.1993.74.2.520 8458765

[B21] Lieber R. L. Runesson E. Einarsson F. Friden J. (2003). Inferior mechanical properties of spastic muscle bundles due to hypertrophic but compromised extracellular matrix material. *Muscle Nerve* 28 464–471. 10.1002/mus.10446 14506719

[B22] Lieber R. L. Woodburn T. M. Friden J. (1991). Muscle damage induced by eccentric contractions of 25% strain. *J. Appl. Physiol.* 70 2498–2507. 10.1152/jappl.1991.70.6.2498 1885443

[B23] Lieber R. L. Yeh Y. Baskin R. J. (1984). Sarcomere length determination using laser diffraction. Effect of beam and fiber diameter. *Biophys. J.* 45 1007–1016. 10.1016/s0006-3495(84)84246-0 6610443 PMC1434983

[B24] Linke W. A. Popov V. I. Pollack G. H. (1994). Passive and active tension in single cardiac myofibrils. *Biophys. J.* 67 782–792. 10.1016/s0006-3495(94)80538-7 7948691 PMC1225421

[B25] Maas H. Baan G. C. Huijing P. A. (2001). Intermuscular interaction via myofascial force transmission: effects of tibialis anterior and extensor hallucis longus length on force transmission from rat extensor digitorum longus muscle. *J. Biomech.* 34 927–940. 10.1016/s0021-9290(01)00055-0 11410176

[B26] Maas H. Jaspers R. T. Baan G. C. Huijing P. A. (2003). Myofascial force transmission between a single muscle head and adjacent tissues: length effects of head III of rat EDL. *J. Appl. Physiol.* 95 2004–2013. 10.1152/japplphysiol.00220.2003 12844495

[B27] Magid A. Law D. J. (1985). Myofibrils bear most of the resting tension in frog skeletal muscle. *Science* 230 1280–1282. 10.1126/science.4071053 4071053

[B28] Maruyama K. (1986). Connectin, an elastic filamentous protein of striated muscle. *Int. Rev. Cytol.* 104 81–114. 10.1016/s0074-7696(08)61924-5 3531066

[B29] Maruyama K. Yoshioka T. Higuchi H. Ohashi K. Kimura S. Natori R. (1985). Connectin filaments link thick filaments and Z lines in frog skeletal muscle as revealed by immunoelectron microscopy. *J. Cell Biol.* 101 2167–2172. 10.1083/jcb.101.6.2167 3905821 PMC2114010

[B30] Mendez J. Keys A. (1960). Density and composition of mammalian muscle. *Metabolism* 9 184–188.

[B31] Meyer G. A. Lieber R. L. (2011). Elucidation of extracellular matrix mechanics from muscle fibers and fiber bundles. *J. Biomech.* 44 771–773. 10.1016/j.jbiomech.2010.10.044 21092966 PMC3042517

[B32] Meyer G. A. Lieber R. L. (2012). Skeletal Muscle Fibrosis Develops in Response to Desmin Deletion. *Am. J. Physiol. Cell Physiol.* 302 C1609–C1620. 10.1152/ajpcell.00441.2011 22442138 PMC3378016

[B33] Meyer G. Lieber R. L. (2018). Muscle fibers bear a larger fraction of passive muscle tension in frogs compared with mice. *J. Exp. Biol.* 221 1–5. 10.1242/jeb.182089 30237238 PMC6262763

[B34] Olsson M. C. Kruger M. Meyer L. H. Ahnlund L. Gransberg L. Linke W. A. (2006). Fibre type-specific increase in passive muscle tension in spinal cord-injured subjects with spasticity. *J. Physiol.* 577 339–352. 10.1113/jphysiol.2006.116749 16931550 PMC2000690

[B35] Powell P. L. Roy R. R. Kanim P. Bello M. A. Edgerton V. R. (1984). Predictability of skeletal muscle tension from architectural determinations in guinea pig hindlimbs. *J. Appl. Physiol.* 57 1715–1721. 10.1152/jappl.1984.57.6.1715 6511546

[B36] Prado L. G. Makarenko I. Andresen C. Kruger M. Opitz C. A. Linke W. A. (2005). Isoform diversity of giant proteins in relation to passive and active contractile properties of rabbit skeletal muscles. *J. Gen. Physiol.* 126 461–480. 10.1085/jgp.200509364 16230467 PMC2266601

[B37] Purslow P. P. Trotter J. A. (1994). The morphology and mechanical properties of endomysium in series-fibred muscles: variations with muscle length. *J. Muscle Res. Cell Motil.* 15 299–308. 7929795 10.1007/BF00123482

[B38] Sacks R. D. Roy R. R. (1982). Architecture of the hindlimb muscles of cats: functional significance. *J. Morphol.* 173 185–195. 10.1002/jmor.1051730206 7120421

[B39] Shah S. B. Davis J. Weisleder N. Kostavassili I. Mcculloch A. D. Ralston E. (2004). Structural and functional roles of desmin in mouse skeletal muscle during passive deformation. *Biophys. J.* 86 2993–3008. 10.1016/s0006-3495(04)74349-0 15111414 PMC1304166

[B40] Smith L. R. Lee K. S. Ward S. R. Chambers H. G. Lieber R. L. (2011). Hamstring contractures in children with spastic cerebral palsy result from a stiffer extracellular matrix and increased in vivo sarcomere length. *J. Physiol.* 589 2625–2639. 10.1113/jphysiol.2010.203364 21486759 PMC3115830

[B41] Takahashi M. Ward S. R. Lieber R. L. (2007). Intraoperative single-site sarcomere length measurement accurately reflects whole-muscle sarcomere length in the rabbit. *J. Hand. Surg. Am.* 32 612–617. 10.1016/j.jhsa.2007.03.002 17481997

[B42] Thacker B. E. Tomiya A. Hulst J. B. Suzuki K. P. Bremner S. N. Gastwirt R. F. (2011). Passive mechanical properties and related proteins change with botulinum neurotoxin A injection of normal skeletal muscle. *J. Orthop. Res.* 30 497–502. 10.1002/jor.21533 21853457 PMC3227753

[B43] Trombitas K. Jin J. P. Granzier H. (1995). The mechanically active domain of titin in cardiac muscle. *Circ. Res.* 77 856–861. 10.1161/01.res.77.4.856 7554133

[B44] Trotter J. A. Purslow P. P. (1992). Functional morphology of the endomysium in series fibered muscles. *J. Morphol.* 212 109–122. 10.1002/jmor.1052120203 1608046

[B45] Trotter J. A. Richmond F. J. Purslow P. P. (1995). Functional morphology and motor control of series-fibered muscles. *Exerc. Sport Sci. Rev.* 23 167–213. 7556350

[B46] Wang K. (1985). Sarcomere-associated cytoskeletal lattices in striated muscle. *Rev. Hypothesis. Cell Muscle Motil.* 6 315–369. 10.1007/978-1-4757-4723-2_10 3888377

[B47] Wang K. Mccarter R. Wright J. Beverly J. Ramirez-Mitchell R. (1991). Regulation of skeletal muscle stiffness and elasticity by titin isoforms: a test of the segmental extension model of resting tension. *Proc. Natl. Acad. Sci. U.S.A.* 88 7101–7105. 10.1073/pnas.88.16.7101 1714586 PMC52241

[B48] Ward S. R. Tomiya A. Regev G. J. Thacker B. E. Benzl R. C. Kim C. W. (2009). Passive mechanical properties of the lumbar multifidus muscle support its role as a stabilizer. *J. Biomech.* 42 1384–1389. 10.1016/j.jbiomech.2008.09.042 19457491 PMC2752430

[B49] Warren C. M. Krzesinski P. R. Greaser M. L. (2003). Vertical agarose gel electrophoresis and electroblotting of high-molecular-weight proteins. *Electrophoresis* 24 1695–1702. 10.1002/elps.200305392 12783444

[B50] Williams P. E. Goldspink G. (1984). Connective tissue changes in immobilised muscle. *J. Anat.* 138(Pt 2), 343–350. 6715254 PMC1164074

[B51] Winters T. M. Takahashi M. Lieber R. L. Ward S. R. (2011). Whole muscle length-tension relationships are accurately modeled as scaled sarcomeres in rabbit hindlimb muscles. *J. Biomech.* 44 109–115. 10.1016/j.jbiomech.2010.08.033 20889156 PMC3003754

